# Does Exonerating an Accused Researcher Restore the Researcher’s Credibility?

**DOI:** 10.1371/journal.pone.0126316

**Published:** 2015-05-13

**Authors:** Tobias Greitemeyer, Christina Sagioglou

**Affiliations:** University of Innsbruck, Innsbruck, Austria; Tilburg University, NETHERLANDS

## Abstract

Scientific misconduct appears to be on the rise. However, an accused researcher may later be exonerated. The present research examines to what extent participants adhere to their attitude toward a researcher who allegedly committed academic misconduct after learning that the researcher is innocent. In two studies, participants in an exoneration and an uncorrected accusation condition learned that the ethics committee of a researcher’s university demanded the retraction of one of the researcher’s articles, whereas participants in a control condition did not receive this information. As intended, this manipulation led to a more favorable attitude toward the researcher in the control compared to the exoneration and the uncorrected accusation conditions (pre-exoneration attitude). Then, participants in the exoneration condition learned that the researcher was exonerated and that the article was not retracted. Participants in the uncorrected accusation and the control condition were not informed about the exoneration. Results revealed that the exoneration effectively worked, in that participants in the exoneration condition had a more favorable attitude (post-exoneration attitude) toward the researcher than did participants in the uncorrected accusation condition. Moreover, the post-exoneration attitude toward the researcher was similar in the exoneration and the control conditions. Finally, in the exoneration condition only, participants’ post-exoneration attitude was more favorable than their pre-exoneration attitude. These findings suggest that an exoneration of an accused researcher restores the researcher’s credibility.

## Introduction

Many scientific disciplines have been plagued by academic misconduct, and it appears that this unhelpful state of affairs is on the rise. In fact, the number of retracted articles due to misconduct, including fraud, duplicate publication, and plagiarism, has dramatically increased in recent years [1,2]. Hence, the goal to detect and eliminate academic misconduct from scientific publications is more important than ever. At times, it is quite clear when there is a case of academic misconduct (e.g., the accused researchers admit that they had reported fraudulent data). At other times, however, the evidence for academic misconduct is less clear-cut.

Consider the case of Jens Förster, a social psychologist who was working at the University of Amsterdam. Förster has been accused of academic misconduct after statistical analyses had suggested that data in some of his published findings had been manipulated. Based on these analyses, further scrutiny of Förster’s data files, and an “inadequate account of the data collection and of the original data”, the Dutch National Board for Scientific Integrity (LOWI) has concluded that data in Förster’s work were manipulated and recommended the retraction of one of his articles. Although the journal editor has later decided to follow this recommendation [3], it is important to keep in mind that the LOWI concluded that it cannot be determined whether Förster had manipulated the data. Förster has denied that he manipulated any data and indicated that he felt like the victim of a witch hunt, although he would not rule out the possibility that his lab assistants manipulated data [4]. In cases like these, there is always a certain risk of accusing a researcher who is in fact innocent. At the time of writing, it is unclear whether Förster has engaged in any academic misconduct; he might be found guilty or he may be fully exonerated. In this paper, we address how researchers, such as Jens Förster, will be perceived if they are later exonerated. Will people perceive them as being completely innocent or will they adhere to their belief that they are guilty?

Abundant research has addressed the extent to which mistaken beliefs persist despite corrective efforts (for a review, [5]). Several lines of work suggest that corrections do not effectively lead to the abandonment of inaccurate beliefs. For example, abundant evidence has demonstrated that even when people clearly understand, believe, and remember that their misperception has been corrected, they still believe in the truth of the misinformation [6–8]. It appears that the misinformation remains available in memory and may more or less automatically affects people’s memory and inferential reasoning. In fact, this so-called continued influence effect is reduced but not eliminated when participants are explicitly warned at the outset about the misinformation effect [9]. Moreover, even multiple retractions fail to eliminate the continued influence effect completely [6]. Likewise, research into the so-called debriefing paradigm, which has also been employed in the present studies, has demonstrated that people persevere in beliefs even after learning that the evidence on which their beliefs were originally based has been disclosed as false [10,11]. For example, Ross and colleagues [12] asked their participants to distinguish between genuine and unauthentic suicide notes. After participants had received false feedback indicating that they had done better or worse than average at the test, they were debriefed about the false nature of the feedback. Despite this invalidation, participants who had received positive feedback believed that they had performed better than average and participants who had received negative feedback believed that they had done worse than average. Overall, it appears that individuals are reluctant to revise their initial beliefs [13,14].

On the other hand, attitudes are not always fundamentally resistant to change. Under some circumstances, they can change after corrective efforts. In fact, research has shown that debriefing procedures do work to some extent. For example, in one study [15] participants in two experimental conditions learned about a scientific finding, whereas participants in a control condition did not. Next, participants in one of the experimental conditions were debriefed that the article had been retracted because of fabricated data. Finally, participants’ belief in the truth of the finding was assessed. Participants in the control condition, who had not previously learned about the finding, were least likely to believe in it, whereas those who were not debriefed about the retraction were most likely to believe in it. Importantly, participants who were debriefed were in between these former groups. Overall, this data pattern suggests that individuals do adjust their beliefs after debriefing, but insufficiently (see also [10]). Other research [16] showed that, compared to a control condition where no rebuttal was presented, the rebuttal of a political misperception significantly reduced the extent to which participants were committed to the false belief. Moreover, receiving a correction from a source that is deemed to be highly trustworthy effectively reduces the impact of erroneous information [17]. Finally, Davies [18] found that participants who neither generated nor were provided with an explanation for an outcome did not exhibit belief perseverance after learning about the fictitious nature of the outcomes. We will discuss this study in more detail in the General Discussion.

## The Present Research

Taken together, it appears that corrections do successfully reduce beliefs in misinformation, although often insufficiently. Hence, we anticipated that a researcher who is accused of academic misconduct but is later exonerated would be perceived less favorably than a researcher who has never been accused of academic misconduct, but more favorably than a researcher who has been accused of academic misconduct without subsequent exoneration. These hypotheses were examined in two studies. For both studies, there were no data exclusions, and all manipulations and all measures analyzed are reported. Participants’ sex did not affect the main dependent measure and, thus, has not been considered further.

### Ethics

In Austria, it is not necessary to get explicit ethical approval if the study conforms to the guidelines of the German Psychological Society. As this is the case for the current research, we consulted the University of Innsbruck's Review Board "Psychologie" and the head of the ethics board provided a waiver of approval. At the beginning of each study, participants read detailed instructions regarding ethical guidelines (i.e., that the data are analyzed anonymously and that they are free to abstain from participation in the study or to withdraw consent to participate at any time without reprisal). They further learned that the submission of responses would be taken as permission to use these in research analysis and in resulting publications. Moreover, no personal or identifying information was collected from the participants. The University of Innsbruck's Review Board "Psychologie" approves this consent procedure.

### Study 1

Participants learned about research concerning the relationship between physical elevation as a metaphor for heightened virtue and its effect on prosociality. We employed an actual article that was later retracted at the request of the author. According to the retraction notice, the data reported are invalid [19]. Participants in the exoneration condition and the uncorrected accusation condition learned that the ethics committee of the authors’ university demanded the retraction of the article, whereas participants in the control condition did not. Following this, participants’ pre-exoneration attitude toward the lead author was assessed. Assuming that the manipulation is successful, participants in the control condition should have a more favorable attitude toward the researcher than participants in the exoneration and uncorrected accusation conditions. Participants in the exoneration condition later learned that the author was exonerated and that the article was not retracted, whereas participants in the uncorrected accusation condition and the control condition did not learn about the exoneration. Then, participants’ post-exoneration attitude toward the researcher was assessed. We anticipated that participants in the uncorrected accusation condition would have a less favorable attitude toward the researcher than participants in the exoneration condition and the control condition, that is, the exoneration should affect participants’ post-exoneration attitudes [10,15,18]. Moreover, we anticipated that participants in the control condition would have a more favorable post-exoneration attitude than participants in the exoneration condition. Taken together, the exoneration of an accused researcher should restore their credibility, but this restoration, however, should be insufficient.

#### Method

One-hundred-and-eighty-five individuals (117 females, 68 males; mean age = 37.9 years, *SD* = 13.3) took part on Amazon Mechanical Turk (MTurk) in exchange for a payment of US $0.25. They were randomly assigned to one of three experimental conditions. There were 68 participants in the exoneration condition, 46 participants in the uncorrected accusation condition, and 71 participants in the control condition. In our previous research on belief perseverance [14] in which we employed the same experimental design as in the present studies, 158 individuals participated. Hence, for both studies, we aimed for at least this number of participants.

At the onset, all participants learned that Lawrence Sanna is a famous social psychologist who works as a researcher and professor at a prestigious university. The research he conducts together with his team explores the relationship between physical height (e.g., riding an ascending escalator) and prosocial behavior. To manipulate the participants’ initial attitude toward the researcher, participants in the exoneration and the uncorrected accusation conditions learned that Sanna was accused of reporting inconclusive data and that the ethics committee of his university demanded the retraction of a scientific article. This part was printed in bold. Participants were then given the following summary of the article:

“The article that was published in 2012 in the Journal of Experimental Social Psychology addressed the effect of bodily height on helping behavior. Sanna and colleagues assumed that based on the metaphorical relationship between heightened virtue and prosociality there would be a causal relationship between physical height and subsequent prosocial behavior. In their studies, they found that people who were going up an escalator donated more money than people who were taking the escalator down. Also, people who were sitting on an elevated level (11 ft.) donated more money and were more empathic than people who were sitting on a lower level (5.5 ft.). In a further study, participants who watched a 5-minute height inducing film clip (e.g., flying above the clouds) showed more cooperative behavior in a subsequent task than did participants who did not watch such a film clip. Participants in the control condition were constantly less helpful than “elevated” participants, but more helpful than “low” participants. According to Sanna and his coauthors these studies provide converging evidence that elevated physical height increases subsequent helping behavior, whereas lowered physical height decreases subsequent helping behavior.”

Participants in the control condition were not informed about the article. Afterwards, participants’ initial attitude toward the researcher was assessed by the following three questions (pre-exoneration attitude: α = .92): “To what extent do you think that Lawrence Sanna is a trustworthy researcher? (anchors: not trustworthy, very trustworthy),” “To what degree do you believe empirical findings that are published by Lawrence Sanna? (anchors: don’t believe at all, believe very much),” and “To what extent should Lawrence Sanna receive financing for his future research? (anchors: no further financing, definitely further financing).” All items were assessed on a scale from −5 to +5.

Afterwards, participants responded to some filler items (e.g., “How important is it for you that your opinion regarding the previously made judgments is confirmed?” and “How much do you generally hold on to your opinions?”) that did not significantly affect the main findings (i.e., the interaction remained significant when using the ratings as covariates and the effect size was very similar). The same applies to Study 2. Then, participants in the exoneration condition learned that it turned out that Sanna and his colleagues were wrongly accused. None of the allegations were confirmed and so, eventually, the article was not retracted. The exoneration was printed in bold. Participants in the uncorrected accusation condition and the control condition did not learn about the exoneration. Then, all participants responded to the same three questions concerning the attitude toward the researcher (post-exoneration attitude: α = .94).

Afterwards, participants were asked what they thought this experiment was trying to study. None of the participants had any correct assumption about the purpose of the study. Participants were also asked whether they were aware of Lawrence Sanna and his research prior to participating in our study, but no one indicated that they had. After the study was over, all participants were thanked and thoroughly debriefed. Specifically, participants were informed that the article by Lawrence Sanna had, indeed, been retracted due to fabricated data. Furthermore, they were given the email address of our laboratory if they had any further questions in regards to this study.

#### Results

**Fig 1:**
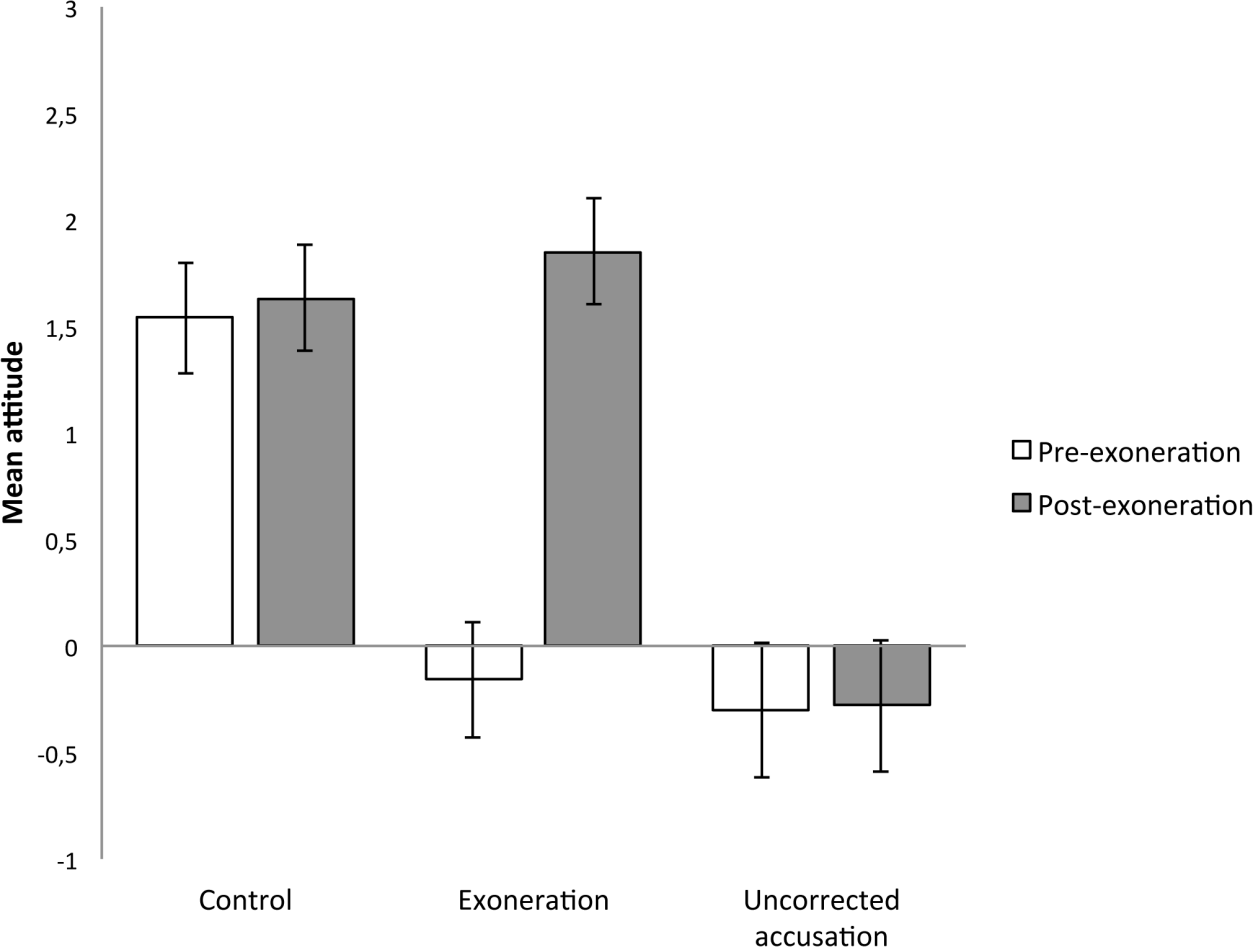
Mean pre- and post-exoneration attitudes as a function of experimental condition (Study 1). Error bars depict two standard errors.

A 3 (experimental condition: exoneration, uncorrected accusation, control) x 2 (attitude: pre- vs. post-exoneration) analysis of variance (ANOVA), with repeated measures on the latter factor, was performed on the data. The interaction was significant, *F*(1, 182) = 25.32, MSE = 40.60, *p* < .001, η_p_^2^ = .22 (see Fig 1). Participants’ pre-exoneration attitude toward the researcher significantly differed across experimental conditions, *F*(2, 182) = 13.91, MSE = 67.16, *p* < .001, η_p_^2^ = .13. A planned contrast showed that participants in the control condition (contrast weight: +2, *M* = +1.54, *SD* = 1.72, 95% CI = [1.02, 2.05]) had a more favorable attitude toward the researcher than participants in the exoneration (contrast weight: -1, *M* = -0.16, *SD* = 2.53, 95% CI = [-0.68, 0.37]) and uncorrected accusation conditions (contrast weight: -1, *M* = -0.30, *SD* = 2.33, 95% CI = [-0.94, 0.34]), *t*(182) = 5.27, *p* < .001. The orthogonal contrast comparing the exoneration (contrast weight: +1) with the uncorrected accusation condition (contrast weight: -1) was not significant, *p* = .739. That is, the experimental manipulation was successful, in that learning about possible academic misconduct led to a negative attitude toward the researcher.

Participants’ post-exoneration attitude toward the researcher also significantly differed across experimental conditions, *F*(2, 182) = 16.43, MSE = 71.26, *p* < .001, η_p_^2^ = .15. A planned contrast showed that participants in the uncorrected accusation condition (contrast weight: -2, *M* = -0.28, *SD* = 2.53, 95% CI = [-0.89, 0.32]) had a less favorable attitude toward the researcher than participants in the exoneration (contrast weight: +1, *M* = +1.85, *SD* = 2.22, 95% CI = [1.35, 2.35]) and the control condition (contrast weight: +1, *M* = +1.63, *SD* = 1.57, 95% CI = [1.14, 2.12]), *t*(182) = 5.71, *p* < .001. Unexpectedly, the orthogonal contrast comparing the exoneration (contrast weight: +1) with the control condition (contrast weight: -1) was not significant, *p* = .536. That is, the exoneration fully restored the researcher’s credibility.

To put it differently, in the exoneration condition, the post-exoneration attitude toward the researcher was more favorable than the pre-exoneration attitude, *F*(1, 67) = 37.99, MSE = 136.67, *p* < .001, η_p_^2^ = .36. In contrast, for both participants in the control condition, *F*(1, 70) = 0.55, MSE = 0.31, *p* = .462, η_p_^2^ = .01, and the uncorrected accusation condition, *F*(1, 45) = 0.02, MSE = 0.00, *p* = .887, η_p_^2^ = .00, the post-exoneration attitude toward the researcher did not significantly differ from the pre-exoneration attitude.

#### Discussion

Study 1 showed that the exoneration effectively corrected participants’ initial beliefs. Whereas participants in the control condition had a more favorable pre-exoneration attitude toward the researcher than participants in the exoneration condition, their post-exoneration attitudes did not significantly differ (if anything, participants in the exoneration condition had a more favorable post-exoneration attitude). In addition, participants in the exoneration condition had a more favorable post-exoneration attitude toward the researcher than participants in the uncorrected accusation condition. Finally, in the exoneration condition, participants’ post-exoneration attitude was more favorable than their pre-exoneration attitude.

As noted above, the finding that the exoneration and the control conditions did not significantly differ was unexpected. We did expect that the exoneration would work in that participants’ attitude toward the researcher should have been more favorable in the exoneration compared to the uncorrected accusation condition. However, we also expected that the exoneration should have worked insufficiently, in that participants’ attitude toward the researcher should be more favorable in the control compared to the exoneration condition. The exoneration condition differed from the control condition in that only the former group of participants learned about an accused researcher who is later exonerated. But it is noteworthy that the two experimental conditions differed in another way: whereas the exoneration participants learned that the accused researcher was the author of an article in an empirical journal, participants in the control condition did not receive this information. That is, the post-exoneration attitude toward the researcher might not be more favorable in the control compared to the exoneration condition because only the latter group of participants explicitly learned about an empirical article that had been published by the researcher. This issue was addressed in Study 2.

### Study 2

Study 2 was a conceptual replication of Study 1, with the following modifications. First, in Study 1, we employed an actual academic misconduct case. Although none of our participants indicated that they were aware of the retraction of the article before participating in our study, we decided to employ a fictitious case in Study 2. Second, and even more importantly, we included an additional control condition. In Study 1, participants in the exoneration and the uncorrected accusation condition explicitly learned that the researcher was an author of a scientific article, whereas participants in the control condition did not. We employed this condition in our design of Study 2 (no-article control condition), but we also included a further control condition in which the participants learned that the researcher was an author of a scientific article (article control condition). Unlike the exoneration and the uncorrected accusation condition, however, no information about possible academic misconduct was given.

With regard to the pre-exoneration attitudes, we anticipated that participants in both control conditions would have a more favorable attitude toward the researcher than participants in the exoneration and uncorrected accusation conditions. Moreover, explicitly learning that a researcher is the author of a scientific article should lead to a favorable attitude toward the researcher in that participants in the article control condition should have a more favorable attitude toward the researcher than participants in the no-article control condition. With regard to the post-exoneration attitude, we anticipated that participants in the uncorrected accusation condition would have a less favorable attitude toward the researcher than participants in the remaining three experimental conditions. That is, an accused researcher who is not exonerated should be negatively perceived by others. Moreover, participants in the no-article control condition should have a less favorable attitude toward the researcher than participants in the exoneration and the article control condition, indicating that being an author of a scientific article positively affects the attitude toward the researcher. Finally, we expected that participants in the article control condition would have a more favorable attitude toward the researcher than participants in the exoneration condition. Overall, such a pattern of findings would suggest that exonerating an accused researcher restores the researcher’s credibility, but insufficiently.

#### Method

Three-hundred-and-fifty-eight individuals (148 females, 210 males; mean age = 32.1 years, *SD* = 10.2) took part on Amazon Mechanical Turk (MTurk) in exchange for a payment of US $0.25. They were randomly assigned to one of four experimental conditions. There were 94 participants in the exoneration condition, 78 participants in the uncorrected accusation condition, 89 participants in the article control condition, and 97 participants in the no-article control condition.

At the onset, participants learned that Robert Miller is a famous social psychologist who works as a researcher and professor at a prestigious university. The research he conducts together with his team explores the relationship between sweet taste and interpersonal attraction to people of the opposite sex. Participants in the exoneration condition and the uncorrected accusation condition learned that Miller was accused of reporting inconclusive data. Therefore, the ethics committee of his university demanded a retraction of a scientific article. This part was printed in bold. Participants in the article control condition and the no-article control condition were not informed about these allegations.

Participants in the exoneration condition, the uncorrected accusation condition, and the article control condition were then given the following summary of the (fictitious) article:

“The article that was published in 2012 in the Journal of Experimental Social Psychology addressed the effect of sweet taste on interpersonal attraction. Miller and colleagues assumed that sweet taste experiences would increase the attraction to people of the opposite sex. Indeed, in their studies they found that heterosexual participants who consumed sweet food felt more attracted to people of the opposite sex. In the first study, 97 participants ate sweet chocolate cookies, whereas 94 other participants ate non-sweet cookies. Afterwards, participants rated photographs of the opposite sex regarding their attractiveness. Results showed that people who ate chocolate cookies perceived the people in the photographs as more attractive than did people who ate the non-sweet cookies. In a second study (243 participants altogether), people consumed either a sweet or a neutral drink. Subsequently, they rated the attractiveness of photographs of people of the same and the opposite sex. This study showed that people who had drunk a sweet drink perceived people of the opposite sex as more attractive than did people who had drunk a neutral drink. The ratings of people of the same sex did not differ. According to Miller and his coauthors, these studies provide converging evidence that sweet taste experiences increase attraction to the opposite sex.”

Participants in the no-article control condition were not informed about the article. Afterwards, the procedure and the methodology were the same as in Study 1 (pre-exoneration attitude α = .90, post-exoneration attitude: α = . pre-exoneration attitude toward the91). However, inasmuch as we used a fictitious researcher and article, we did not assess whether participants learned about Robert Miller and his research before participating in our study. At the end, participants were debriefed about the fictitious nature of the researcher Robert Miller and of the scientific findings that identified a relationship between sweet taste and interpersonal attraction.

#### Results

**Fig 2:**
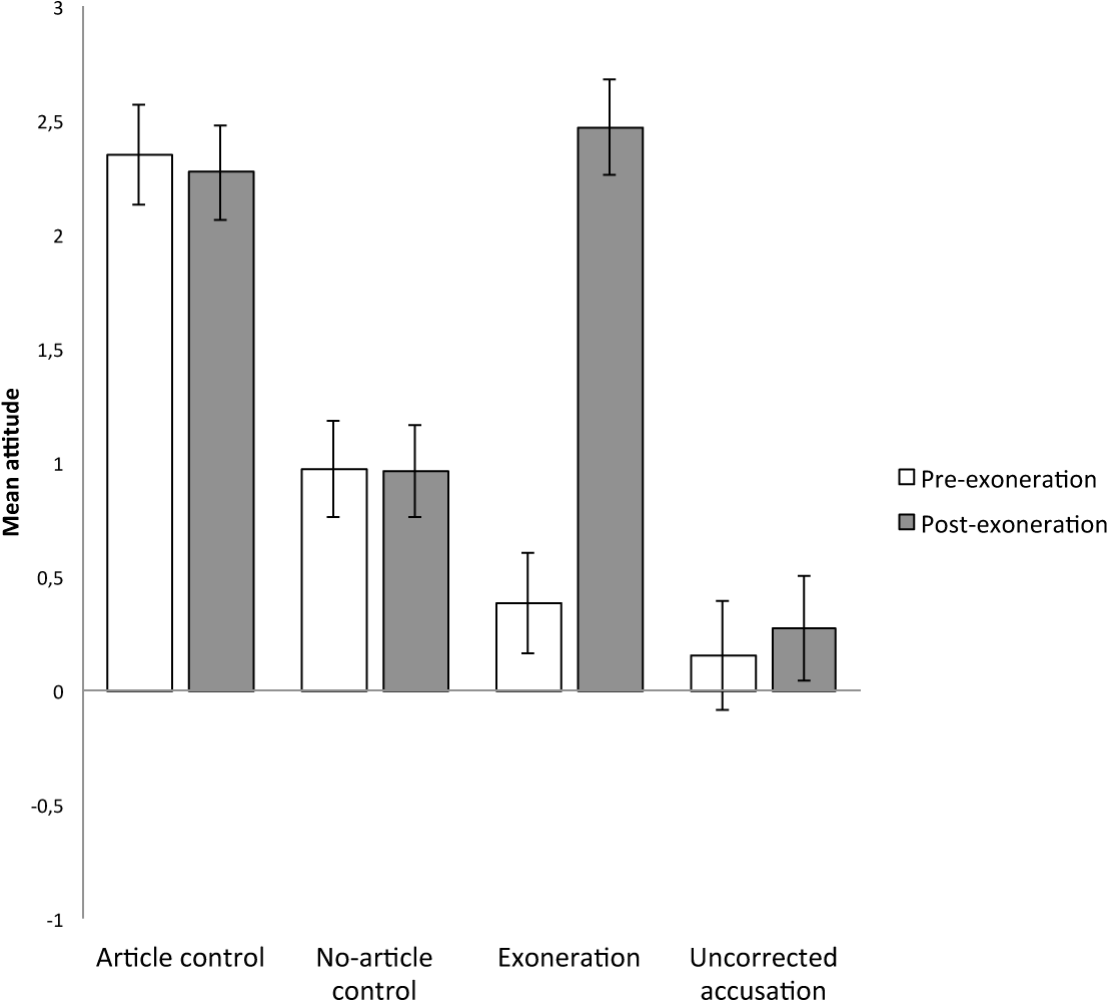
Mean pre- and post-exoneration attitudes as a function of experimental condition (Study 2). Error bars depict two standard errors.

A 4 (experimental condition: exoneration, uncorrected accusation, article control, no-article control) x 2 (attitude: pre- vs. post-exoneration) ANOVA, with repeated measures on the latter factor, revealed a significant interaction, *F*(3, 354) = 47.27, MSE = 50.31, *p* < .001, η_p_^2^ = .29 (see Fig 2). Participants’ pre-exoneration attitude toward the researcher significantly differed across experimental conditions, *F*(3, 354) = 19.29, MSE = 84.65, *p* < .001, η_p_^2^ = .14. A planned contrast showed that participants in the article control condition (contrast weight: +1, *M* = +2.35, *SD* = 1.37, 95% CI = [2.06, 2.64]) and the no-article control condition (contrast weight: +1, *M* = +0.97, *SD* = 1.69, 95% CI = [0.63, 1.31]) had a more favorable pre-exoneration attitude toward the researcher than participants in the exoneration (contrast weight: -1, *M* = +0.38, *SD* = 2.49, 95% CI = [-0.13, 0.89]) and the uncorrected accusation condition (contrast weight: -1, *M* = +0.15, *SD* = 2.65, 95% CI = [-0.45, 0.75]), *t*(354) = 6.27, *p* < .001. The first orthogonal contrast comparing the article control condition (contrast weight: +1) with the no-article control condition (contrast weight: -1) was significant, *t*(354) = 4.49, *p* < .001, indicating that being an author of a scientific article positively affected the attitude toward the researcher. The second orthogonal contrast comparing the exoneration condition (contrast weight: +1) with the uncorrected accusation condition (contrast weight: -1) was not significant, *t*(354) = 0.73, *p* = .467. Overall, the experimental manipulation was successful.

Participants’ post-exoneration attitude toward the researcher also significantly differed across experimental conditions, *F*(3, 354) = 24.34, MSE = 96.26, *p* < .001, η_p_^2^ = .17. A planned contrast showed that participants in the uncorrected accusation condition (contrast weight: -3, *M* = +0.27, *SD* = 2.48, 95% CI = [-0.29, 0.82]) had a less favorable attitude toward the researcher than participants in the exoneration condition (contrast weight: +1, *M* = +2.47, *SD* = 2.15, 95% CI = [2.03, 2.91]), the article control condition (contrast weight: +1, *M* = +2.27, *SD* = 1.41, 95% CI = [1.97, 2.57]), and the no-article control condition (contrast weight: +1, *M* = +0.96, *SD* = 1.83, 95% CI = [0.59, 1.32]), *t*(354) = 6.42, *p* < .001. The first orthogonal contrast comparing the no-article control condition (contrast weight: -2) with the exoneration (contrast weight: +1) and the article control conditions (contrast weight: +1) was significant, *t*(354) = 5.67, *p* < .001, indicating again that being an author of a scientific article positively affected the attitude toward the researcher. Unexpectedly, the second orthogonal contrast comparing the exoneration (contrast weight: -1) with the article control condition (contrast weight: +1) was not significant, *t*(354) = 0.66, *p* = .508. Overall, these analyses suggest that being an author of a scientific article increases the credibility of the researcher and that exonerating an accused researcher fully restores the researcher’s credibility.

To put it differently, in the exoneration condition, the post-exoneration attitude toward the researcher was more favorable than the pre-exoneration attitude, *F*(1, 93) = 57.55, MSE = 204.34, *p* < .001, η_p_^2^ = .38. In contrast, in the article control condition, *F*(1, 88) = 2.54, MSE = 0.25, *p* = .114, η_p_^2^ = .03, no-article control condition, *F*(1, 96) = 0.51, MSE = 0.01, *p* = .822, η_p_^2^ = .00, and the uncorrected accusation condition, *F*(1, 77) = 1.94, MSE = 0.52, *p* = .168, η_p_^2^ = .03, the post-exoneration attitude toward the researcher did not significantly differ from the pre-exoneration attitude.

#### Discussion

Study 2 showed that participants did not maintain their attitude toward an accused researcher after learning about corrective evidence. In fact, participants in the exoneration condition had a more favorable post-exoneration attitude toward the researcher than participants in the uncorrected accusation and the no-article control conditions. Moreover, the exoneration condition did not significantly differ from the article control condition. Finally, only in the exoneration condition, participants’ post-exoneration attitude was more favorable than their pre-exoneration attitude. In sum, the exoneration was successful, in that it fully restored the researcher’s credibility.

Note that the data pattern of Study 2 is not fully consistent with Study 1, where the exoneration condition did not differ from the (no-article) control condition. We will return to this issue in the General Discussion. Nevertheless, both studies converge in that exonerating an accused researcher restores the researcher’s credibility.

## General discussion

The present research suggests that people do abandon their attitude toward an accused researcher after learning that the researcher has been exonerated. In both studies, participants in the exoneration condition had a more favorable attitude toward the researcher than participants in the uncorrected accusation condition. Moreover, in the exoneration condition, participants’ post-exoneration attitude was more favorable than their pre-exoneration attitude. These findings are in line with previous research [10,15,18] showing that exoneration procedures do affect post-exoneration beliefs. Unexpectedly, however, the post-exoneration attitude toward the researcher was very similar in the exoneration and the control condition. That is, the exoneration restores the researcher’s credibility.

Note, however, that there is an important inconsistency in the present findings. In Study 2, participants in the exoneration condition had a more favorable post-exoneration attitude toward the researcher than participants in the no-article control condition. In contrast, in Study 1, in which a no-article control condition was also employed, participants’ post-exoneration attitude in the exoneration and the control condition did not differ significantly. It is noteworthy that participants in the exoneration condition explicitly learned that the accused researcher was the author of an article, whereas participants in the (no article) control condition did not. As Study 2 showed, being the author of a scientific article positively affects the attitude toward the researcher in that participants in the article control condition had a more favorable attitude toward the researcher than participants in the no-article control condition. This finding suggests that the exoneration completely restored the researcher’s credibility in Study 2, whereas in Study 1 the exoneration procedures affected participants’ attitudes insufficiently (relative to what could be expected had the control condition clarified that the researcher was the author of a scientific paper). Yet, in Study 1 participants in the exoneration condition did tend to have a more favorable attitude toward the researcher than participants in the control condition. If the sample size was bigger and given greater statistical power, results might have revealed this finding to be significant. Moreover, both studies converge in that the exoneration led to a more favorable post-exoneration compared to pre-exoneration attitude. That is, participants did adjust their attitude toward the researcher after exoneration.

Why didn’t participants stick to their initial attitude toward the researcher? First, it is important to note that the manipulation of the accusation worked in that learning about academic misconduct led to a negative attitude toward the researcher. As noted in the introduction, many previous studies have shown that people tend to maintain their beliefs even after learning that the evidence on which the beliefs were originally based has been discredited (for a review, [5]). In contrast, in the present studies there were no perseverance effects. There are a number of possible explanations for this unexpected finding.

That participants did not persevere in their beliefs might be due to the credibility of the source of the correcting feedback. As noted in the Introduction, Guillory and Geraci [17] found that perseverance effects are considerably reduced when people receive a correction from a credible source. Their studies suggest that erroneous information is often not corrected because people do not believe corrections. So it may be that our participants did not cling to their initial attitude toward the researcher because they were convinced that the exoneration was accurate. Note also that the accusation of academic misconduct that was given to participants in our studies was rather vague (i.e., participants learned that the researchers were accused of reporting inconclusive data) so that the ethics committee’s decision to demand a retraction might have been questioned.

Previous research has shown that attributional processes underlie belief perseverance [10,15,20,21]. That is, once people form a belief they engage in causal processing that fit the evidence. These explanations in turn continue to imply that the initial belief is correct even after the evidential basis for the belief has been discredited. Although people generate causal explanations spontaneously [20], belief perseverance is greater when people are explicitly asked to generate explanations that fit the evidence. For instance, Anderson and colleagues [10] found that participants who were induced to generate explanations for an outcome showed more perseverance than did participants who were not explicitly induced to do so. Likewise, Davies [18] showed that participants who were asked to generate explanations for an outcome were more likely to continue to believe in the outcome after learning about discrediting evidence than did participants in a control condition who were not encouraged to generate explanations (participants who were given explanations provided by others were in-between). In fact, in the control condition, no significant belief perseverance was observed. In the present studies, we did not explicitly ask our participants to generate explanations as to why the researcher committed academic misconduct. Although it can be assumed that some participants did spontaneously generate explanations that fit the evidence [20], explicitly inducing participants to generate explanations would certainly increase the proportion of participants who engage in causal thinking, and, in turn, would increase the amount of belief perseverance.

In this respect, it is important to note that our samples were comprised of community members, and most (if not all) of them were not working at a research university. One reason why researchers commit scientific misconduct is certainly the pressure to publish. People working in academia should be more familiar with the pressure to publish than those not working within academia and thus it may be that they are more able to generate explanations why an accused researcher committed academic misconduct and, as a consequence, they should be more likely to cling to the initial belief that the researcher committed academic misconduct. It is also possible that people working in academia are particularly likely to condemn academic misconduct, which also leads to the hypothesis that an exoneration works less well for an academic sample of participants. Testing this possibility is an important avenue for future research.

A further limitation involves the recruitment of participants via an online questionnaire. Some readers may wonder to what extent participants were diligent in reading the instructions. In our previous research using online-samples [22], we administered an item manipulation check to verify attentive participation [23]. However, because less than one percent of participants failed this item manipulation check, we abstained from including such a measure in the present studies. Nevertheless, we acknowledge that a replication of our findings employing paper and pencil measures is certainly useful. Finally, there is a chance of having provoked demand effects. Participants in our samples were aware that the study in which they participated was carried out by researchers who—they might have assumed—expect or wish an exoneration to fully restore an accused researcher’s credibility.

## Conclusion

Detecting academic misconduct is an important endeavor that benefits the credibility of scientific research. But it is also important to consider how responding to academic misconduct affects the accused researcher. In particular, if a case of alleged academic misconduct is not clear-cut, then there is always the risk of inaccurate accusations. At least, as the present studies document, it appears that the exoneration of an accused researcher restores the researcher’s credibility.

## Supporting Information

S1 File SPSS data file for Study 1. (SAV)

S2 File SPSS data file for Study 2. (SAV)
